# Carbon Dots: Opportunities and Challenges in Cancer Therapy

**DOI:** 10.3390/pharmaceutics15031019

**Published:** 2023-03-22

**Authors:** Tanima Bhattacharya, Gye Hwa Shin, Jun Tae Kim

**Affiliations:** 1 Department of Food and Nutrition, Kyung Hee University, Seoul 02447, Republic of Korea; 2 Department of Food and Nutrition, Kunsan National University, Gunsan 54150, Republic of Korea; 3 BioNanocomposite Research Center, Kyung Hee University, Seoul 02447, Republic of Korea

**Keywords:** carbon dots, surface chemistry, cancer theranostic, bio-imaging

## Abstract

Recently, carbon dots (CDs) have been actively studied and reported for their various properties. In particular, the specific characteristics of carbon dots have been considered as a possible technique for cancer diagnosis and therapy. This is also a cutting-edge technology that offers fresh ideas for treating various disorders. Though carbon dots are still in their infancy and have not yet shown their value to society, their discovery has already resulted in some noteworthy advancements. The application of CDs indicates conversion in natural imaging. Photography using CDs has demonstrated extraordinary appropriateness in bio-imaging, the discovery of novel drugs, the delivery of targeted genes, bio-sensing, photodynamic therapy, and diagnosis. This review seeks to provide a comprehensive understanding of CDs, including their benefits, characteristics, applications, and mode of action. In this overview, many CD design strategies will be highlighted. In addition, we will discuss numerous studies on cytotoxic testing to demonstrate the safety of CDs. The current study will address the production method, mechanism, ongoing research, and application of CDs in cancer diagnosis and therapy.

## 1. Introduction

Cancer has emerged as the major cause of health problems in the world because of its high incidence and death rate. The overall 5-year survival rate of cancer patients is still quite low, despite outstanding progress in cancer treatment. In 2018, there were roughly 9.6 million cancer-related fatalities and 18 million new cancer cases; there will be 22 million cancer cases annually by the end of the next two decades. Lung cancer will claim the lives of more than 350 individuals per day, which is 2.5 times more than CRC, the second biggest cause of cancer death, and more than breast, prostate, and pancreatic cancers combined. In 2022, smoking-related fatalities will account for about 105,840 of the 130,180 lung cancer deaths (81%) and another 3650 deaths will result from secondhand smoke. If classified individually, the other 20,700 non-smoking-related lung cancer deaths would be the ninth most common cancer death cause for both sexes combined [1]. New medications and methods must be created to treat cancer precisely and effectively [2]. Nanotheranostics that combine diagnosis and therapy onto a single nanoplatform are gaining popularity for cancer treatment [3]. The combination of diagnosis and therapy can gather more specific data, allowing for precise, high-sensitivity, and low-interference cancer treatment [4]. The integration can also track tumor metastasis and recurrence for high therapeutic efficacy. Consequently, a wide range of nanotheranostic agents are available based on two or more imaging techniques such as fluorescence (FL)/photoacoustic (PA) [5], FL/magnetic resonance (MR) [5], MR/positron emission computed tomography (PET) [6], and FL/MR/PET, and therapeutic modalities such as chemotherapy (CHT)/radiotherapy (RT), gene therapy (GT)/photodynamic therapy (PDT), CHT/photo thermal therapy (PTT), and PDT/PTT/CHT [7,8,9,10,11]. Although these methods have become more popular, each form of nanotheranostic agent has particular characteristics and inherent limits [12]. Inorganic nanoparticles such as up-conversion nanoparticles, transition metal chalcogenide, or selenide nanoparticles frequently exhibit special physicochemical features that enable usage in concurrent imaging and therapy of cancer. Long-term safety of heavy metal conjugated nanoparticles is a severe challenge for researchers, thus restricting their usage in theranostic applications. Utilizing fewer components to create innovative, safe, and biocompatible “one for all” theranostic features for clinical multimodal imaging and treatment modalities is still a significant problem in the field of research on low-toxicity materials. Carbon dots (CDs) have received a lot of attention over the past 10 years in a variety of fields, including bio-imaging, photocatalysis, light-emitting diodes, and the promotion of plant growth because of their advantageous electronic, mechanical, optical, thermal, and biocompatible properties [13]. When CDs were unintentionally found in 2004 by Xu and colleagues, CDs or carbon quantum dots (CQDs) were used because their spectral characteristics are similar to the then-popular silicon quantum dots (QDs). There are now two categories for CD preparation methods: top-down and bottom-up [14].

CDs have been acknowledged as wise choices for the diagnosis of cancer [15]. CDs have been investigated for cancer diagnosis by PA imaging, MR imaging, and others in addition to using FL imaging [16]. Some CDs also naturally possess anticancer characteristics, which take part in PDT and PTT [17]. PDT typically involves the use of photosensitizers [18] to produce reactive oxygen species (ROSs) under the right lighting conditions, which are then used to cause oxidation reactions with biological macromolecules in cancer cells, leading to cell toxicity; PTT [19] typically involves the use of photothermal agents to absorb photon energy before releasing heat in the tumor site, which causes irreparable damage to cancer cells. In PDT and PTT, the light illumination can be spatially adjusted to irradiate only the tumor lesion without harming healthy tissues. When compared to conventional chemotherapy and radiotherapy, the selectivity provided by PDT and PTT can dramatically lower systemic toxicity. As a result, CDs with inherent photo-theranostic capabilities have demonstrated significant promise for use in the treatment of tumors [20,21]. The main objective of the present review is to highlight synthesis techniques of CDs and the prospects of CDs as nanotheranostic agents for anticancer applications. Although much research relates to CD synthesis and applications in bio-imaging cancer cells, we are dealing with more recent trends of cancer treatments with the help of conjugated CDs for imaging, nanotheranostics, surface chemistry, mechanism of actions, and challenges faced. Research gaps are also highlighted with new ideas to overcome the barriers of the application of CDs in cancer therapy.

## 2. CDs: Synthesis, Properties, and Mechanism

**Figure 1:**
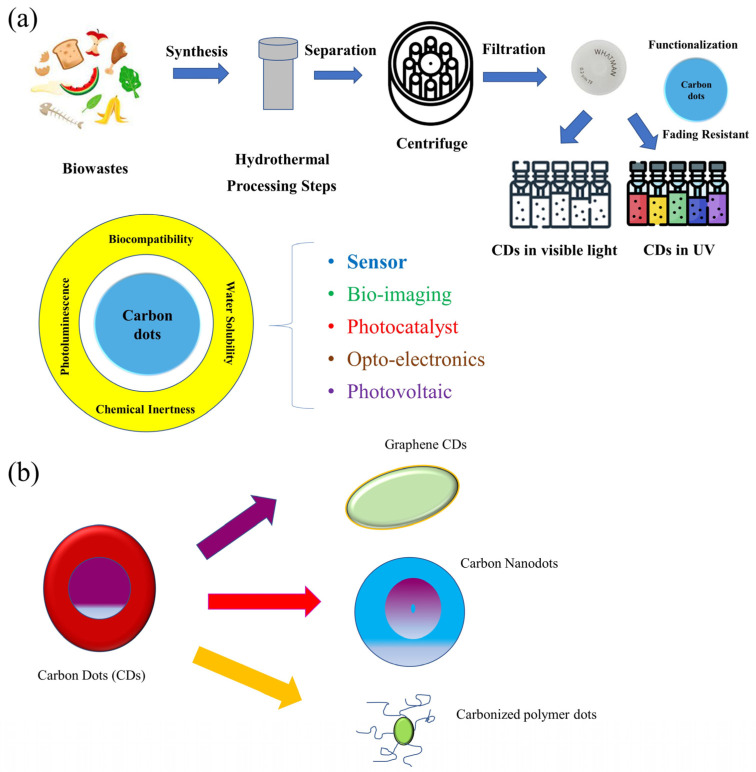
Synthesis techniques (**a**) and types (**b**) of CDs.

CDs can be prepared in a variety of practical and efficient ways [22]. One of these is the bottom-up approach, where carbon hydrates are used as a carbon source to react with solvents under certain synthesis circumstances, and another common technique is a top-down method that depends on the fragmentation of carbon allotropes, such as nanotubes, graphene, and fullerenes [23]. The most popular and straightforward preparation techniques, including hydrothermal or solvothermal treatment and microwave irradiation, use little to no extra reagents [24]. Laser ablation, chemical ablation, and electrochemical carbonization are some further uncommon techniques that produce acceptable results. Figure 1a depicts the synthesis methods coupled with numerous pathways that lead to varied uses, demonstrating the adaptability of CDs. CDs can be divided into three types based on their structural characteristics viz. graphene quantum dots (GQDs), carbon nanodots (CNDs), and carbonized polymer dots (CPDs), as shown in Figure 1b. Despite differences in structure, size, and surface functional groups, all classes of CDs show equivalent photoluminescence (PL) features [25]. GQDs are made up of a few layers of graphene with chemical groups on the edges that are the result of reactants derived from carbon sources or from later reactions [26]. Conversely, CNDs have a spherical form [27] and can have an amorphous structure in carbon nanoparticles (CNPs) or a crystalline graphite-like lattice in CDs. Organic polymer chains and a carbon core make up the new and growing fluorescent nanomaterials known as CPDs. CPDs are often created via condensation, cross-linking, or mild carbonization from monomers or polymers. When compared to conventional CDs, CPDs have a low level of carbonization––possibly even zero percent. Hence, the processes of condensation and cross-linking are necessary for the synthesis of CPDs. Because of their particular chemical makeup, CPDs exhibit distinctive qualities like strong emission, high yield, and high oxygen levels that guarantee excellent water solubility. Furthermore, the polymer chain structure of CPDs enables simple functionalization of CPDs through covalent bonding or blending with inorganic substances, polymers, and functional molecules. The functionalized CPDs have potential uses in medication delivery, fluorescent displays, and photoelectric devices. CDs can either be cross-linked/aggregated linear polymers or chains of polymer gathered around a sphere of carbon [28,29]. 

The bottom-up strategy carbonizes organic molecules or biomass using a range of technologies, including the template method [30], pyrolysis [31], microwave [32], solvothermal, etc., to produce emitting CDs. On the other hand, the top-down strategy is preparing smaller luminous CDs with large bulk carbons, such as graphite, carbon fiber, carbon nanotubes, graphene, etc., by utilizing laser ablation, electrochemistry, arc discharge, and other technologies [22,33]. CQDs, GQDs, CPDs, and CNDs are the four basic types of CDs [31,34]. Traditional II-VI QDs’ metal toxicity can be successfully mitigated by CDs, making them useful as very stable, biocompatible fluorescence probes and biological tracers in vivo [35]. The most important characterizations the researchers focused on for the synthesized or market available CDs are their optical properties, photoluminescence, morphological study properties, cytotoxicity, antioxidant, antibacterial, and anti-cancer activities [23].

The PL of these nanomaterials show various lifetime, intensity, and emission wavelength depending on the structure, size, and surface group. Ananthanarayanan et al. [36] thoroughly investigated the relationship between properties and morphology. Their research revealed that wavelength increases with the number and size of surface groups. It is interesting to note that the authors have shown that various forms of heteroatom doping can cause a red or blue shift in the emission spectrum. Compared to QDs, CDs can exhibit low cytotoxicity, making them suitable for in vivo studies depending on the type of ligand used for passivation.

Most of the CD types introduced so far exhibit broad UV-vis absorption peaks and PL emission peaks in the blue-to-green visual spectrum [37]. In particular, the challenge in developing CDs for in vivo studies is to ensure that CDs emit with a high quantum yield (QY) in the infrared (635–700 nm) [38]. To do this, it is necessary to understand the process underlying the fascinating PL properties of CDs. The exact process underlying CD’s remarkable quality has been the subject of numerous studies, but none of these mechanisms have yet been demonstrated. Currently, the most widespread theory is that surface groups at the edge of the CDs, rather than the same size as in QDs, are responsible for the PL emission [39]. After the transition of aromatic sp2 C-C bond, the absorption of CDs in the UV-vis spectrum reveals a peak at 230–280 nm [40]. According to Zheng and his group [41], a shoulder between 300 and 550 nm is attributed to the n-* transition of C=O/C=N and C-O/C-N bonds or other related groups, where GQDs with altered size are linked to shifts at the maximum of the emission spectrum. These findings lead to the conclusion that quantum confinement is not the only factor influencing the PL properties of CDs, but is nonetheless important to consider when defining the emission and absorption spectra of these unique materials. The theory underlying the absorption and emission spectra of CDs is still in its infancy, making it difficult to determine the relationship between the maximum emission and the QY with the size of the CDs, their population, and their surface functioning. However, some straightforward guidelines have been developed. For instance, nitrogen doping to improve the QY of CDs has been discovered, and numerous studies have been made based on this discovery [42,43,44,45,46,47,48].

Photo-induced electron transfer is an important mechanism for CDs in cell apoptosis imaging. Based on the strong interaction between Annexin V and phosphatidylserine, a non-toxic Annexin V-conjugated CD probe was created by Mahani et al. [49]. The photoinduced electron transfer between the CDs and the tyrosine and tryptophan in the annexin structure caused the fluorescence of the CDs to be quenched in the absence of phosphatidylserine. When it binds to the phosphatidylserines on the outer layer of apoptotic cells, it can activate. The created probe can be employed for phosphatidylserine detection and real-time imaging of cell apoptosis and was biocompatible, stable, affordable, and selective.

## 3. CDs as Cancer Detectors

Quantum dots (QDs), a heterogeneous family of manufactured nanoparticles with distinctive optical and chemical properties, have emerged as one of the most interesting developments in label technology. QDs have a wide range of possible uses from energy to medicine with high specificity and sensitivity [50]. Compared to QDs, fluorescent CDs outperform organic dyes in terms of hydrophilicity, biocompatibility, ease of manufacture, and lesser toxicity. These factors are what lead to CDs being regarded as effective for cancer detection. In addition, different colors of QDs can be concurrently activated with just a single light source with minimum spectral overlaps, which provides a considerable advantage for the combinatorial detection of target molecules. QDs can be utilized for cancer detection and treatment with great specificity when coupled with diagnostic (such as optical) and therapeutic (such as anticancer) substances [50]. QDs have decreased background and good tissue penetration as deep as 1 cm making them appropriate for diagnosing lymph node metastases [51]. Imaging probes can be created using fluorescent CDs. CDs have been combined with metals like gadolinium, which lessen their toxicity to organs and also stop their leakage [52]. The majority of the fluorescence sensors for Fe^3+^ detection being developed now are based on CDs. Cancer and other illnesses can arise when Fe^3+^ levels are abnormal. Research was conducted where Glutathione (GSH) was mixed with CDs to observe the fluorescence efficacy [53], and as the concentration of GSH is correlated with cancer progression, the research leads to a new paradigm for cancer cell detection. Elevated GSH levels result in the reduction of oxidative stress observed in cancer cells, whereas deficiency leads to cancer proliferation and progression. Additionally, it was discovered that GSH could increase the fluorescence of a CDs and Fe^3+^ mixed solution (CDs/Fe^3+^), and that this ability could be used to effectively separate malignant cells from normal cells based on the difference in GSH content between the two types of cells. The potential of CDs/Fe^3+^ for imaging-guided precision cancer diagnosis is more significant. They also observed the elevated fluorescence signals of CDs/Fe^3+^ in the tumor site in vivo following intravenous injection [54].

Green fluorescence CDs and a related probe with fluorescence activation were developed for the visual detection of cancer [55,56,57]. Folic acid was conjugated with CDs to create images of cancer cells. With turn-on fluorescence, the probe was able to identify cells that were positive for the folate receptor (FR) [58]. In a different work, folic acid-conjugated fluorescent CDs that can bind to FR were created. With FR, these CDs were able to distinguish between healthy cells and A549 adenocarcinoma human basal epithelial cancer cells and displayed remarkable biocompatibility.

Wu et al. developed an assay for the identification of human cervical cancer cells and breast cancer cells employing modified CDs as Electro Chemi Luminescent (ECL) sensors and graphene as a signal amplifier [59]. The sensors were created in the following ways. First, the electrode was modified by covalently attaching poly(allylamine hydrochloride) (PAH), which has an amino functional, to graphene. The CDs-Ag composite was then applied to the electrode’s surface. The electrode was made from surface modified graphene to prepare a label-free and sensitive detector of cancer cells, which provided a larger surface area.

Folic acid was further conjugated with cysteine via a carbodiimide-mediated wet chemistry method between the carboxylic acid end groups on folic acid and the amine groups on cysteine after the amino acid cysteine, which contains thiols, was linked to the CDs-Ag surface [59]. The sensitivity and selectivity of the sensor were significantly impacted by each of the modifying processes as follows: (1) the metal shell on CDs enhanced the electron transport between CDs-Ag and graphene, (2) folic acid could specifically and effectively target folate receptors with high affinity, allowing them to serve as a link between cancer cells and the electrode, and (3) the high surface area and strong conductivity of the graphene conjugation may make electron transport easier [59]. The manufactured electrode could specifically identify the surface of human cervical cancer cells (HeLa) and human breast cancer cells (MCF-7) by targeting the folate receptor because of the specificity and affinity of folic acid. Because cancer cells blocked the surface of the CDs-Ag nanocomposite and kept them from coming into contact with the ECL core actant N2S2O8, the ECL signal was noticeably reduced in the presence of cancer cells. The detection threshold was determined to 10 cells per mL at 3 s [59]. For instance, Phuong and coworkers [60] created a selective and sensitive nanotheranostic nanoplatform based on pH-responsive cross-linked CDs integrated with titanium oxide; TiO_2_ (C-CD/TiO_2_) for the purpose of diagnosing tumors using the precise targeting capabilities of the tumor cell membrane and nuclei. Another method for diagnosing cancer, in addition to directly imaging cancer cells, is to in vivo image the biomarkers in tumors. For instance, a group of scientists described a method for bio-imaging cathepsin B (CTSB), one of the most promising biomarkers for a variety of malignant tumors that allowed for the effective early detection of tumors [61]. In the study, researchers created a particular type of amine-rich CDs and then covalently assembled the nucleolin targeting recognition nucleic acid aptamer AS1411 and a CTSB-cleavable peptide substrate that was tethered with chlorin e6 (Ce6), enabling a ratio metric nanoprobe of AS1411-Ce6-CDs that was both cancer-targeting and CTSB stimulus-responsive [61]. FRET is completed, and the ratiometric fluorescence response to CTSB is achieved. Incorporating a cancer-targeting recognition moiety would enable a robust ratiometric fluorescence technique to report CTSB activity. Table 1 summarized different precursors, techniques, surface functionalization, target moiety, and applications of CDs as a cancer theranostic.

## 4. CDs as Cancer Bio-Imaging Agents

CDs have been used as contrast agents for in vivo optical imaging because of their new photo physical features [20,66]. The most used method for optical imaging is direct PL imaging using light irradiation. The structure of cells or tissues can be recreated using the emitted photon of an imaging agent in down-conversion or multiphoton excited-up conversion fluorescence [67,68]. However, the Abbe criterion’s diffraction limit for light places a restriction on common PL imaging with ordinary fluorescence microscopy. In order to get around the diffraction limit, two main strategies are used: (i.) single molecule localization-based imaging, which includes stochastic optical reconstruction microscopy (STORM) and photo activated localization microscopy, and (ii.) patterned illumination-based imaging, which includes stimulated emission depletion (STED) microscopy and structured illumination microscopy (SIM). Closely clustered fluorescent particles are resolved for single molecule localization-based imaging by stochastically turning on and off each particle’s signal, and the centroid of the on-state particle is then mathematically determined in each imaging frame. The super-resolution PL image can be recreated under certain circumstances by combining several iterations. Afterglow imaging is regarded as another great method for bioimaging in addition to direct PL imaging. Because of the unique delay luminescence, afterglow imaging using phosphorescence and thermally activated delayed fluorescence (TADF) can often reduce the noise of background photo excited auto fluorescence [69]. Similar to that, Cathodoluminescence (CL) is also used as a contrast imaging technique. The CL imaging can be used as a special biomolecular sensor with ultrahigh sensitivity and provide distinctive bio-imaging without photo-excited auto fluorescence from the background because the CL emission is the result of a chemical reaction without photoexcitation [70]. Following that, other tactics have been created to further achieve targeting cancer cells by bio-imaging. Researchers discovered the effect of CD accumulation in tumor areas early on and further refined uptake accumulation focused imaging [71]. As nanotechnologies advanced, CDs underwent further development to enable tumor targeting imaging with logical design. Some examples include stimulus-responsive imaging evoked by the novel charge and pH in the tumor microenvironment and in vivo biomarker imaging through interactions between CDs and various biomolecules [72]. Additionally, numerous studies have identified and approved uniquely targeted CDs, which are cancer cell markers for a variety of specific malignancies, allowing both their unique use in self-targeted bio-imaging and potential methods for cancer diagnosis [73]. The discovery of CDs is considered a powerful new class of nanoprobes and has been demonstrated to be utilized as a contrast agent in numerous bio-imaging models [74]. The ideal CDs for bio-imaging would have high PL quantum yield (QY) [75], long-wavelength PL emission [76,77], little toxicity or nontoxicity [78], and renal cleavability [79]. This would allow for the reasonable use of intrinsic CDs in both in vitro and in vivo biological system visualization [80]. Early on, CDs have homogenous penetration and dispersion across all cells, giving both normal and malignant cells the same uptake accumulation [81]. However, the enhanced permeability and retention (EPR) effect has led various studies to notice the unique buildup of CDs in tumor tissues. Su et al. observed, for instance, that CDs preferentially accumulated at the tumor site and were effectively cleared by the kidneys [55]. In their research, a brand-new class of Hafnium-doped CDs (Hf-CDs) demonstrated preferential targeted tumor accumulation capability with important benefits like robust stability, good biocompatibility, excellent water solubility, and exceptional computed tomography (CT) contrast performance, which enables the CDs, in particular CT/fluorescence imaging, to be used for orthotropic liver cancer. Researchers discovered during their tests that the Hf-CDs may aggregate at the tumor site, enabling quick bio-imaging, which suggests a simple and flexible multimodal imaging technique. Additionally, these researchers use a variety of techniques to improve the unique cancer cells’ uptake accumulation. For long-term mitochondria-targeting cellular imaging using CDs, researchers developed a biocompatible nanoplatform. They also improved the magnetic field-enhanced cellular uptake functions to boost the unique accumulation [82]. This study used surface modification to create a biocompatible nanoplatform using a magnetic mesoporous silica nanoparticle (Fe_3_O_4_@mSiO_2_) conjugation of fluorescent CDs with triphenylphosphine (TPP) [82]. Additionally, studies showed that in a static magnetic field, the mice lung cancer cell (A549) and human foreskin fibroblast (HFF) cell lines’ cellular uptake efficiency may be quickly improved [82]. These unique CD accumulations in tumor areas served as the foundation for targeted uptake and opened the way [61]. In addition to direct uptake accumulation, these stimulus-responsive techniques have also been investigated to accomplish tailored uptake of CDs depending on the unique microenvironment in tumor locations [82]. For instance, the zwitterionic CDs can easily form bio conjugates with a variety of biomolecules via the interaction between the biomolecules and their carboxylic moieties. They shed their anionic component, and leave a positive charge on their surface when they interact with the microenvironment of cancer cells, enhancing their targeted uptake in cancer cells for a variety of tailored bio-imaging techniques. Based on this, Sri et al. created a specific class of zwitterionic CDs and described their targeted bio-imaging for the human tongue and pharyngeal cancer cell lines [83].

The DNA aptamer AS1411 modified CDs with polyethyleneimine (PEI) as the connecting bridge were used by Kong et al. to develop an effective detected and targeted nanosystem [62]. Researchers demonstrated in their studies that MCF-7 cells took up the CDs-PEI-AS1411 nanosystem more readily than L929 cells did, demonstrating the extremely selective nature of the ability to detect nucleolin-positive cells. The pH-responsive interaction was suggested as a further factor to boost the cellular uptake selectivity of free CDs in addition to the charge interaction [62]. Researchers created the hydrophobic dopa-decyl CDs (D-CDs) and the zwitterionic-formed CDs (Z-CDs) to target the nucleus and the hydrophobic regions of the cell membrane, respectively [60]. The fluorescence “off” state at physiological pH and the fluorescence “on” state in acidic cancer cells were used for tumor-selective biosensors by the disruption of the Förster resonance energy transfer (FRET) with the boronate ester linkages between the D-CD and Z-CD [60]. The C-CD/TiO_2_ demonstrated outstanding targeted bio-imaging and therapeutic capabilities by effectively ablating tumors in the in vivo tumor model with up regulation of the pro-apoptotic markers in the tumor [60]. While this was going on, a few researchers created a particular type of CD derived from aconitic acid (AA-CDs) and showed that the AA-CDs could recognize folic acid (FA) with specificity, which led to fluorescence quenching [84]. In their research, scientists created a method for FA analysis that is sensitive, with a detection limit of 40 nM. They also created one type of fluorescent nanoprobe (FA-AA-CDs) through the conjugated interaction between FA and AA-CDs, enabling them to be used as fluorescence turn-on nanoprobes for targeted imaging of cancer cells [84]. Hela, SMMC-7721, and A549 cells are models of cancer cells with varying amounts of folate receptors (FRs) expression. FA-AA-CDs demonstrated specifically targeted imaging of cancer cells with the matching association between the PL intensity of these cells and their FRs expression levels [84]. Similar to this, Das et al. reported the ACD-GNP nanohybrid, which included an anionic CDs (ACD) protected gold nanoparticle (GNP), as a nanoprobe for imaging glutathione (GSH) [65]. Based on the GSH-triggered change between the fluorescent indicator ACD and the GNP, these researchers suggested that the ACD-GNP nanohybrid might selectively detect GSH [65]. The ACD-GNP hybrid achieved selective imaging of cancer cells because it was more sensitive and selective to GSH than other bio thiols. Furthermore, a specific type of dual-emission CD was created by researchers confirming their capacity for ratio metric GSH sensing in cancer cells [85]. These CDs could be used as an efficient tool for targeted imaging of cancer cells due to their intrinsic ratio metric fluorescence displacement for GSH sensing [80]. Similar to these widespread techniques for detecting a biomarker directly, methods for selectively identifying substances like antibodies or cancer cell metabolites have also been developed. In order to specifically feel and photograph hyaluronidase (HAase), Gao et al. created one type of turn-on fluorescent nanoprobes of P-CDs/HA-Doxorubicin by electrostatic assembly of PEI-modified CDs (P-CDs) and Hyaluronic acid (HA)-conjugated doxorubicin (Dox) [86]. In their research, the P-CDs/HADox demonstrated low PL emission in a physiological environment. The conjugate was able to enter cancer cells only because of the activation of HAase by using their overexpressed HA to CD44 receptors, leading to an accurate distinction of cancer cells and sensitive assay of HAase [86]. In order to create biocompatible nanoprobes for cancer cells, Demir’s group linked one type of CD with molecularly imprinted polymers (MIPs). The glucuronic acid (GlcA), an epitope of hyaluronan and a biomarker for certain malignancies, was specifically recognized by the researchers by using the emission of CDs as an internal light source for photopolymerization. Demir’s group demonstrated hyaluronan targeting imaging and used CD-based nanocomposites to specifically identify human cervical carcinoma [87].

CDs have been proven to be an effective targeted imaging agent for cancer diagnosis, but their clinical application continues to present significant challenges due to their complicated chemical makeup and potential toxicity [88]. Recently, several different types of CDs have been demonstrated to be distinctively self-targeting to cancer cells, opening the door to promising uses in other fields. For instance, using a direct pyrolysis approach with D-glucose and L-aspartic acid, Zheng et al. designed self-targeted CDs (CD-Asp) with the ability to target brain cancer glioma [89]. Without the need for an additional targeting molecule, the CD-Asp with tunable PL emission demonstrated selective targeted activity toward C6 glioma cells [89]. In general, there are two main mechanisms that may let different substances cross the BBB: transporter-mediated transport and receptor-mediated transport. The high density of the glucose transporter (GLUT-1) on the BBB and in brain tumors confers the ability to target brain tumors by facilitating glucose metabolism. Nonetheless, ASCT2 is a crucial L-isomer-selective transporter across the BBB for the high affinity substrates L-glutamine and L-asparagine. The functional groups from the same reactants (glucose, L-ASP, and/or L-Glu) should be present in all of the CDs created in the that work so that they can pass over the BBB using the GLUT-1 and ACT2 transporters. With these CDs, fluorescence imaging was seen in the brain sites for that reason. RGD, a tripeptide made of L-arginine, Glycine, and L-aspartic Acid, is a well-known glioma-targeting substance that binds to the immature endothelial cells’ αVβ3 integrin [89]. Different drugs can often cross the BBB using one of two main mechanisms: transporter- or receptor-mediated transports. Because the glucose transporters facilitate glucose metabolism, the glucose transporter (GLUT-1) has the ability to target brain tumors. L-glutamine (L-Glu) and L-asparagine (L-Asp) serve as high-affinity substrates for ASCT2, a crucial L-isomer-selective transporter across the BBB [90]. Researchers found that CDs prepared from glucose, L-aspartic acid, and/or L-glutamate consisting of the reactant functional groups (glucose, L-aspartic acid, and/or L-glutamate) could assist them in crossing the BBB through the GLUT-1 and ASCT2 transporters. RGD, a tripeptide made up of L-arginine, Glycine, and L-aspartic Acid, was a popular glioma-targeting substance that bound to RVBeta3 integrin on immature endothelial cells. The targeting function of CD-Asp was therefore inferred to have come from the development of RGD-like functional groups on the CDs’ edge, which were created and formed from D-glucose and L-Asp. Similar to this, Li et al. produced a number of self-targeted CDs (LAAM TC-CDs) that functionalized with several paired -carboxyl and amino groups and demonstrated their specific accumulation in tumors [91]. The LAAM TC-CDs displayed bright PL emission in their experiment, with an NIR PL emission peak at roughly 700 nm. Researchers suggested that the LAAM TC-CDs could enter cancer cells via interacting with human L-type amino acid transporter 1 (LAT1), and they supported this speculative mechanism with six lines based on several tests: (1) uptake of LAAM TC-CDs was reduced by pretreatment with LAT1 inhibitor BCH; (2) LAAM TC-CD uptake was reduced by LAT1 deletion; (3) lentiviral transduction improved cellular uptake of LAAM TC-CDs by overexpressing LAT1 in HeLa cells; (4) the number of LAAM TC-CDs in various cell lines related to LAT1 expression level, and the LAT1 expression level in cancer cells was higher than normal cells; (5) uptake of LAAM TC-CDs in vivo was promoted by overexpression of LAT1 in tumors; and (6) pretreatment with Leu reduced the amount of LAAM TC-CDs accumulated in tumors [91]. Researchers also achieved NIR fluorescence and photoacoustic imaging for different cancers using functionalized CDs that could load aromatic pharmaceuticals through “π” stacking interactions, as well as tailored drug delivery for chemotherapeutics to the tumors. With these outstanding bio-imaging results, CDs show considerable promise for the clinical diagnosis of cancer. An extensive survey by a group of reviewers [92] revealed that use of CDs in the clinical setting showed they performed exceptionally well for directing precision surgery for papillary thyroid cancer. Researchers assessed the use of CDs as lymphatic tracers in their reports when doing bilateral and complete thyroidectomies for papillary thyroid cancer. The associated results demonstrated that the CDs can clearly identify the Central District lymph nodes and separate thyroid tissue from the surrounding lymphoid adipose tissue, hence lowering the risk of parathyroid gland injury during thyroid cancer. As a result, the research has validated the ability of CDs to achieve targeted cancer bioimaging and approved their tremendous potential for clinic applications in the future [92]. Table 2 summarizes some examples of precursors, technique, size, and the quantum yield of various CDs.

## 5. CDs as Cancer Nanomedicine

In recent years, several advancements have been made in the creation of CDs for PDT and PTT [93]. When used in PDT, the CDs kill cancer cells using ROSs created from ambient oxygen while being excited by light of the appropriate wavelength. Here, a multistate sensitization mechanism is attained by CDs. Pheophytin powders were used to create NIR light-emitting CDs, which display significant in singlet oxygen production and have the potential to be used in the treatment of cancer [94]. PTT, a different form of phototherapy, uses photo thermal agents, which can produce heat by absorbing photon energy and killing cancer cells, and, as a result, NIR-emitting CDs with high photo thermal conversion effects, broad absorbance, and strong fluorescence emission were created [95]. The PTT dramatically slowed the growth of the tumor after an in vivo injection of CDs and increased the lifespan of the mice [95]. PDT and PTT have some benefits in the treatment of cancer, but there are still significant challenges in their practical use. For instance, the hypoxic environment in tumors restricts the therapeutic effectiveness of PDT, and the use of PTT invariably results in harm to the normal tissue surrounding the tumor. In order to overcome these difficulties, specific CD-based therapy is crucial. Long-term studies have been conducted on the selective delivery of anticancer medications, such as EPR-based tumor therapy [96], but anticancer drugs are not suitable for clinical use because they are unable to deliver treatment in a targeted manner to the tumor. Additionally, CD receptor-mediated treatment techniques have recently been developed to overcome the abovementioned difficulties [97]. After being surface-functionalized with certain ligands, CDs can precisely bind to receptors that are overexpressed on cancer cell membranes for target specificity [97]. For drug delivery, the receptor-based modification of CDs is crucial. There are several studies that showed greater in vivo cellular absorption of the CDs during cancer therapy. A targeted ligand called hyaluronic acid (HA) binds to CD44, which is overexpressed on different tumor cells [98]. When administered intravenously, HA-modified CDs (HA-CDs), which were created by hydrothermal treatment with branched poly (ethylene mine) and citric acid, have been shown to accumulate in tumor tissue. The antitumor efficacy of HA-CDs was seen after loading with DOX in two distinct tumor models, providing it with a promising future for use in targeted cancer therapy [98]. Although earlier research on receptor-based tumor therapy saw success, mounting evidence indicates that this approach has much significance. Since the majority of receptors are present in both normal and cancer cells, there are very few receptors that are only upregulated on the membrane of cancer cells. The same ligand may not be effective against all cancer cell types.

Specific carrier transporters that are differentially strongly expressed in cancer cells are used in one promising method of tumor specific theranostic. There are 12 transmembrane domains that make up large neutral amino acid transporters 1 (LAT1), which is primarily in charge of transporting neutral amino acids into cells, making up the permeation route [99]. The 12 transmembrane domains form a sulfur bond with the heavy glycoprotein subunit, which makes LAT1’s location on the plasma membrane more stable. Only a small number of organs, including the placenta, BBB, spleen, testis, and colon express LAT1 in normal tissue. To date, four LATs have been discovered. LAT2 was divided based on how closely its sequence matched that of LAT1. In some particular malignancies, LAT3 and LAT4 seem to play a significant role as transporters [99].

For instance, prostate cancer patients had elevated levels of LAT3 expression. LAT4 is discovered to be expressed in the proximal tubule of the kidney, thick ascending limb epithelial cells, and the basolateral membrane of the small intestine. LAT1 is the LAT that has been the subject of the most research. LAT1 inhibitors, including 2-aminobicyclo-2,2,1-heptane-2-carboxylic acid (BCH), have been utilized to treat cancer using LAT1 as a target in prior research. As BCH’s lack of potency and specificity, it has not been successful in chemotherapy. BCH has the ability to inhibit all four LATs [99].

Researchers recently created the LAAM TC-CDs in their lab by hydrothermally processing TAAQ and citric acid in water solution [100]. Regardless of their sources and locations, LAAM TC-CDs function as carriers and make it possible to deliver drugs to tumor tissue. The intravenous injection of the LAAM TC-CDs for various tumor chemotherapies was successful. LAAM TC-CDs, in contrast to conventional CDs, have the ability to deliver therapeutic medicines to tumors only because they act as inhibitors at the tumor site. The LAAM TC-CDs demonstrated the ability to deliver topotecan (TPTC) to Hela and A549 tumors specifically, which resulted in increased chemotherapeutic efficacy. More importantly, the BBB expresses LAT1, which has proved a significant barrier to the transport of drugs to brain tumors. Finding medicines or drug carriers with the potential to penetrate the BBB has remained a significant barrier up to now for the treatment of brain cancer. Traditional ligand-based targeted therapy has not been very successful at delivering drugs to the brain. Fortunately, LAAM TC-CDs can interact with brain tumors by penetrating the BBB [100].

**Figure 2:**
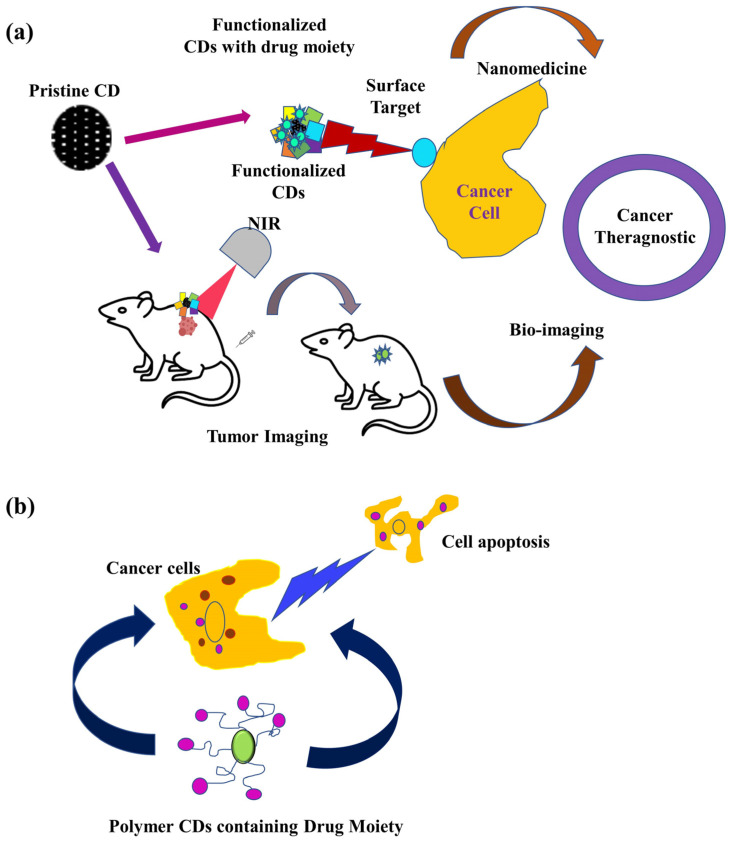
CDs as cancer theranostic NIR bio-imaging (**a**) and apoptosis in cancer cell death (**b**) [52].

It has been shown that LAAM TC-CDs have the ability to deliver TPTC to U87 tumors and perform bioimaging. Through their interaction with LAT1, a target overexpressed in both the BBB and tumor cells, LAAM TC-CDs may successfully treat brain tumors [91]. Figure 2 shows a schematic representation of CDs as nanotheranostic for cancer treatment and a mechanistic approach of CDs in drug delivery by apoptosis in cancer cells.

## 6. Challenges and Opportunities of CDs: Safety Issues

Today, QDs and CDs are frequently used as sensors, probes, and imaging devices [101,102]. Bioconjugation of QDs is also widely used for drug delivery and treatment of devastating diseases, such as cancer [103]. However, bioconjugation of QDs can make some cell delivery difficult because some materials used to make QDs are cytotoxic [104]. The metabolism and excretion of QDs are still unclear, which causes toxicity in the body [105]. Some important limitations restricting its use in biological systems include toxicity [106,107]. The development of harmless QDs such as biowaste based CDs, ZnO-QDs, GQDs, and other CDs prepared from natural materials like milk and fruit skin could overcome this problem [108]. Biowaste resources have also been widely used as low-cost, renewable resources with advantages in biodegradability, biocompatibility, and availability [109]. The increasing interest in using natural resources in CD synthesis using hydrothermal carbonization approaches in recent years requires a minimal experimental setup [110,111,112]. Additionally, there is relatively little information on the regulatory status of QDs and CDs in the literature.

According to nanodots, the chemical composition of the surface could dramatically alter the surface’s physical characteristics, which could have an impact on their PL properties. Nanodots in CG were additionally used with imaging cells that have greater imaging sensitivity than semiconductor toxicity-possible QDs. The in vitro outcomes showed the potent antitumor properties, presenting them as prospective substitutes for imaging and biological applications due to the effects of these nanodot purposes. QDs and CDs are a type of nanoparticle that have undergone three generations of evolution. First-generation NPs had a short circulation period and a large hepatosplenic accumulation because they were not specific to the targeted tissue and were quickly engulfed by the immune system. The idea of stealth was first presented by second-generation NPs. They are protected from the immune system, have a higher water solubility, and are less likely to aggregate when coated with polymer chains like PEG. However, the Enhanced Permeability and Retention (EPR) effect can cause these NPs to assemble in the tumor in specific situations, such as cancer imaging [113]. With a leaky and chaotic vascular network and little to no lymphatic drainage, this condition happens in solid tumors. NPs can gather in tumor sites due to increased vascular permeability and a lack of lymphatic drainage. Unfortunately, this effect is diverse and varies significantly depending on the tumor, the patient, and the stage of the disease. Third-generation NPs were further functionalized with various ligands, including proteins, peptides, nucleic acids, etc., enabling active targeting. These adjustments, nevertheless, sometimes cause fluorescence quenching, and thus need to be carefully thought out.

Near-infrared (NIR) emitting QDs are of particular interest. In fact, NIR light is less absorbed and penetrates tissues deeper, up to several centimeters. Additionally, due to a decreased absorption coefficient from skin and fatty tissue, as well as from oxygenated and deoxygenated blood, tissue autofluorescence is decreased in the NIR window, particularly between 650 and 950 nm. NIR QDs are therefore very intriguing for bioimaging, particularly for imaging malignancy during surgical treatment [113]. Researchers are now studying 3D spheroid cancer models for a better understanding of QD penetration compared to NP nanotheranostics.

Additional research is necessary to create CDs with adequate excitement and emission in the red/near-infrared range, which significantly reduces their value and usefulness performances in biomedical treatments and tests [75]. However, the absence of a thorough manual for streamlining and industrializing the creation of CDs is felt more than ever before and is based on green chemistry methods and biowaste resources. Future CDs with biological potentials are still being considered, with an emphasis on the difficulties in industrial development considering the effect of raw material temperatures, reaction time, pH level, heteroatom co-doping, and other factors on material cytotoxicity (optical, structural) and other aspects of surface passivation characteristics of CDs (biocompatibility, tunability, stability, and catalytic activity). The control of emission color and intensity as well as the enhancement of their surface passivation must also be considered, and other important factors that must also be taken into account include the stabilization of their optical features [9]. Notably, careful consideration should be given to the PL, chemiluminescence, and absorption mechanisms as well as the related photophysical processes of photothermal conversion and ROS generation [44].

We did a brief survey on the toxicity of QDs in potential biomedical applications [114,115]. When working with a large volume of scientific documentation, meta-analysis is a useful method for applying data from the literature. There is literature that describes a meta-analysis study on the toxicity of Cd-based QDs that used random forest regression models to examine the data. According to the authors, a number of surface characteristics, such as shell composition, ligand and surface modification, QD diameter and type of test, and exposure time to biological environments are all closely related to the toxicity of QDs [116]. Although receiving a lot of attention and being used in preclinical settings, one significant unresolved concern with QDs is their potential toxicity. According to certain theories, the physicochemical characteristics of QDs, such as their size, surface charge, ligand type, and interactions with other molecules already present in biological media, can be used to explain why they are poisonous [117].

**Figure 3:**
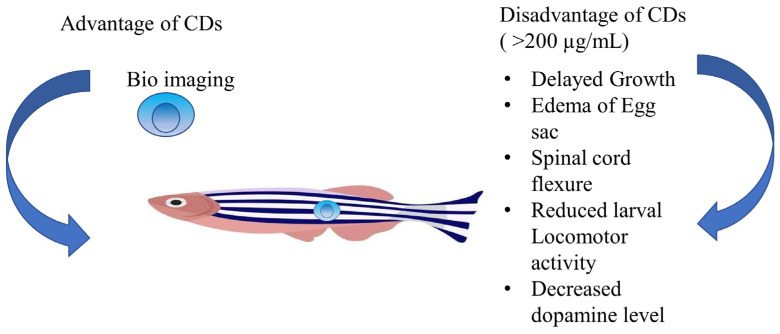
Overview of advantage and disadvantage of CDs in Zebra fish model [122].

L-cysteine (Cys) capped CdTe QDs were created in an aqueous media in an intriguing piece of research. Based on tests with HeLa cancer cell lines, this study hypothesized that the capping agent lowered cytotoxicity [118]. It was strangely discovered that comparing cytotoxicity of CdSe/CdS QDs based on particle concentrations was quite challenging [119]. Moreover, it has been discovered that QDs with high cytotoxicity quickly disintegrate at endosomal pH, releasing Cd (II). Cytotoxicity of CdSe, CdTe, and InP was based on four QD formulations, including CdSe/CdS/ZnS QDs that have been modified with mercaptopropionic acid (CdSe-MPA) and CdSe/CdS/ZnS QDs encapsulated in PEGylated phospholipid (CdSe-Phos), and they were investigated. Surprisingly, two cancer cells—neuroblastoma (SH-SY5Y) and stomach adenocarcinoma (BGC-823)—exhibited various cytotoxic responses [120]. This study thereby brings vital insights to the reality that a combination of factors from the particle compositions and the level of cellular absorption determine the toxicity of QDs rather than being simply dependent on one factor. Thus, the important role of carbon quantum dots of natural sources or biowaste precursors comes from this, where the cells viability was reported at as much as 90% [120]. Here, a zebra fish model was selected, where the feeds were based on spermidine and ammonium nitrate CDs. In order to assess the subacute/subchronic toxicity effects of CDs, adult fish were given oral exposure to CDs at low doses over an extended period of time. Researchers examined changes in the bodyweights of the adult fish and assessed their fecundities across extended feeding periods in order to assess these impacts [121]. Almost 95% of the zebrafish embryos in the fish embryo acute toxicity test (FET) survived exposure to 100 ppm of CDs. Increasing the dose to 500 ppm only caused 40% of embryos to perish. Compared to other metallic QDs, CDs offer significantly greater biocompatibility. There are reports that CDs prepared from kiwi fruits, pear, and avocado had no toxicity on zebra fish [122]. Liu et al. [122] observed that if the concentration exceeds 200 µg/mL of CDs derived from sugarcane molasses in the zebra fish model, they can lead to reduced larval locomotor activity, decreased dopamine level, neurotoxicity, edema of the egg sac, and delayed growth. Figure 3 gives an overview of the advantages and disadvantages of CDs in the zebra fish model.

## 7. Future Perspective and Conclusive Remarks

Despite the fact that QDs offer a wide range of applications in cell imaging and discoveries, toxicity and pharmacological issues are preventing advancements in cancer therapy and detection due to considerable colloidal and metal insecurity [123]. Although these issues might not prevent the development of applications in vitro, they significantly hinder the use of in vivo malignant growth imaging in humans. The development of more resilient and toxic-free nanomaterials for in vivo photo thermal therapy usage (therapy and diagnosis) is essential for future in vivo applications [124]. The discipline is currently developing swiftly, and significant breakthroughs are anticipated in the near future. However, it is important to consider difficulties like the degradation of the covering shell brought on by changing the quantum dots. According to their composition, sizes, surface coatings, and valences, various efforts should be made to build distinctive QDs in order to reduce toxicity and improve detection performance. Nonetheless, it is important to consider problems like coating shell degradation brought on by QDs.

Keeping in mind the properties of QDs such as (i.) the large surface area and excellent conductivity of CD substrates, (ii.) the appropriate design of sensory components, and (iii.) efficient electrochemical measurement techniques, Wei et al. [125] fabricated the use of QDs in a portable sweat sensor to multiplex the detection of a cardiovascular health biomarkers sensor. In order to enhance the functional activity of cardio myocytes, another group of researchers created CD loaded SF/PLA nanofibrous bioactive scaffolds in their study [125]. On the outside of silk fibroin/poly lactic acid (SF/PLA) scaffolds, the manufactured CDs are equally dispersed and admirably cross-linked. When compared to SF/PLA scaffolds without CDs, the synthesized bioactive scaffolds demonstrated improved compressive modulus and favorable swelling values. According to the findings, manufactured kiwi fruit peel-CDs (KFP-CDs) are nontoxic to both healthy cells and malignant cells (up to 150 g mL^−1^), which is crucial for the secure and long-term advancement of cellular imaging. The KFP-CDs could also be employed as a cell-labeling agent for MSCs, breast cancer cells, and thyroid cancer cells for imaging both in vitro and in vivo. In light of this, the research has paved the road for the future development of biowaste materials into novel, pollution-free goods and usages. Biowaste may now be converted into an environmentally acceptable substance for use in biomedicine [126].

The development of charge-convertible cisplatin (IV) prodrug-loaded CDs (CDs-Pt(IV)@PEG-(PAH/DMMA)) that may react to the extracellular milieu of the tumor for imaging-guided drug administration with improved cancer treatment effectiveness has been achieved by Feng et al. [127]. A number of benefits include multicolor bio-imaging, prolonged blood circulation time, effective accumulation at the tumor site, enhanced internalization by cancer cells, facilitated endosome escape, and controlled intracellular drug release. CDs-Pt(IV)@PEG-(PAH/DMMA) has a charge-changing property that changes from a negative charge at a normal physiological condition (pH 7.4) to a positive one at a tumor extracellular microenvironment (pH 6.8) [127]. The charge-convertible CDs’ strong tumor-inhibition efficacy and the few side effects were further shown by in vivo tests, demonstrating the CDs’ potential as a smart drug nano carrier with improved therapeutic benefits. The work offered a plan to encourage prospective clinical use of CDs in the treatment of cancer [112]. 

**Figure 4:**
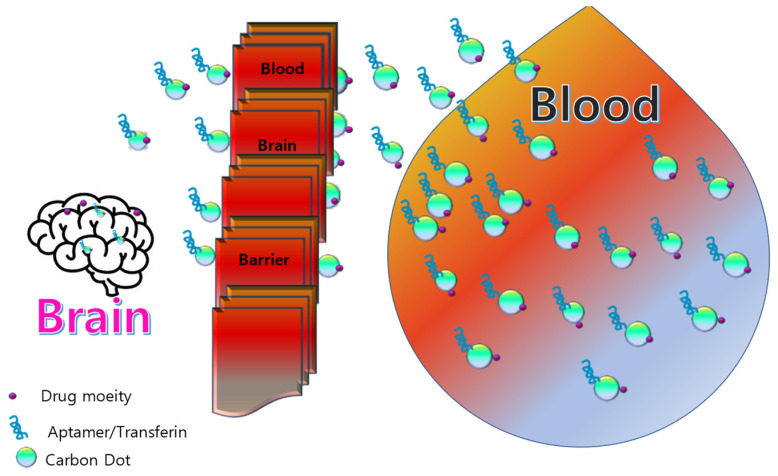
Schematic representation of CDs as promising nano carrier for drug delivery across blood brain barrier [90].

While doing an extensive survey, the reviewers want to propose some ideas about why there are no vast studies on the blood brain barrier of CDs for treatments of brain diseases like brain cancers. Certain characteristics of NPs have been shown to allow medication delivery into the CNS by overcoming the BBB (Figure 4) [90]. Due to their low cytotoxicity and superior biocompatibility compared to metal-based NPs, CDs have drawn a lot of attention. Additionally, CDs’ simple synthesis methods and high surface-to-volume ratio enable them to have a high drug-loading capacity. CD distribution can be monitored in both in vitro and in vivo investigations because of their superior PL. Furthermore, certain CDs may be able to traverse the BBB thanks to some beneficial surface characteristics, including low charge and amphiphilicity. Both in vitro and in vivo models have been created to test whether CDs and CD-conjugated derivatives may traverse the BBB. In a one-pot hydrothermal process, Lu et al. created nitrogen-doped CDs (N-CDs) and examined their capacity to penetrate the blood-brain barrier (BBB) using an in vitro model consisting of rat microvascular endothelial cells and astrocytes [128]. N-CDs’ potent blue PL under UV illumination proved that they were transported through the BBB in a concentration-dependent manner. A notable example of an in vitro investigation is the trans well model used in the N-CDs study. The fundamental drawback of this biomimetic model is that it is still a poor mimic of the BBB in a live animal, despite having tunable parameters and few changes. Zebrafish and mice are frequently used as in vivo models for the BBB-related investigations to get around this restriction [129]. There are reports on nanomaterial bio-imaging for cardiac treatment, but the toxicity is a great factor for those, and, thus, CDs can lead to a pathway for identifying asymptomatic cardiac tumor presence if researchers can work more on the QY and PL properties of CDs. Furthermore, the advancement of contemporary nanomedicine is being significantly aided by artificial intelligence (AI). Through its effective learning ability based on a large database, machine learning has been used to assist in the synthesis and analysis of nanomaterials, including CDs. Recently, it was claimed that highly luminous, photos table, and environmentally stable CDs may be synthesized with the use of machine learning, greatly improving their optical characteristics. Additionally, compared to traditional statistical methods, machine learning has demonstrated a greater ability to forecast, assess, and identify features of CDs, which will advance the study of nanomaterials and nanomedicine. Overall, the chemistry and machine learning combo offers a fresh approach to designing and producing nanomaterials in the future. Due to their intrusiveness and accessibility into previously unreachable regions inside the human body as well as technological advancements in actuation, sensing, and fabrication at the micro- and nanoscales, miniature untethered medical robots have recently drawn more interest. However, the key challenges to the development of medical robotics are propulsion and biocompatibility. In order to overcome these difficulties, Singh’s group [130] outlined a class of sperm cell-driven microrobots known as sperm bots, which are naturally biocompatible and use the sperm cells’ flagellar movement for propulsion. Diverse biological micro-swimmer populations (such as bacteria and algae) were extensively explored in addition to current advancements in biological cell-driven biohybrid systems. A bacteria-driven biohybrid micro-swimmer’s propulsion performance was improved by Singh et al. [130] by the development of a stable and specific bacteria-attachment technology. Other bio hybrid microdevices and systems can also use these surface patterning and attachment approaches. Therefore, once these two challenges are overcome, biological cell-driven medical robots appear to be a great “vehicle” for achieving a quick targeted distribution of CD-based nano medicine in the near future.

## 8. Conclusions

CDs are an appealing luminous nanomaterial that might make it possible to create a workable optical imaging platform because of its organic and biocompatible nature, tunable PL, and adaptable surface functionalization. The inability to tune the PL into the NIR region while simultaneously getting a high PL QY is a significant drawback of C-dots. For NIR fluorescence imaging, long-wavelength excitation is necessary to improve tissue penetration and boost resolution at the same time. Therefore, a thorough analysis of PL processes for various types of CDs are necessary. In this present review, we discussed photoluminescent mechanisms with the principle of photo-induced electron transfer. Here, the safety issues and challenges of CDs as a cancer theranostic are also discussed. Few ideas were proposed for better utility of CDs by surface functionalization. Recent studies on CDs have shown a variety of their physicochemical characteristics through proof-of-concept tests. These characteristics matter for biological imaging. Although CD applications have received positive news, the precise mechanism of cellular uptake and the long-term toxicological implications have yet to be determined. The pharmacokinetics and bio-distribution of CDs are complicated because they depend on a variety of variables, including their morphology, physiochemical characteristics, surface chemistry, and formulation.

## Figures and Tables

**Table 1:** Precursors, technique, surface functionalization, target moiety, and applications of CDs as nanomedicine.

Precursors	Technique	Surface Functionalized Material	Target Moiety	Type of Nanotheranostic	Reference
Folic Acid	Wet chemistry	Cysteine	Cervical cancer & breast cancer cell lines	Nanomedicines	[59]
N/A		Hafnium	Orthotopic Liver Cancer	Bio-imaging	[57]
DNA aptamer AS1411	Hydrothermal	Polyethylene diamine	Breast cancer cell lines	pH responsive drug delivery & bio-imaging agent	[62]
Amine rich	Pyrolysis	Chlorine (Ce6)	Various malignant tumors	Bio-imaging	[63]
Citric Acid	Microwave assisted	PEI & SiRNA	Gastric Cancer Cells	Drug Delivery	[64]
Amino acid		Gold Nanoparticles	Cancer cells	Glutathione sensing	[65]

**Table 2:** Precursor, doping agent, color, synthesis technique, size, and quantum yield (QY) of various CDs.

Precursor	Dopant	Color	Synthesis Technique	Size (nm)	QY (%)	Ref.
Cadmium perchlorate hydrate	Chitosan and B –lymphocyte antigen CD20	Green at 254 nm Orange at 365 nm	Hydrothermal	2–3	N/A	[15]
Hyaluronic acid	Carboxymethyl chitosan	Green	Hydrothermal	4–5	11.64	[17]
Folic acid	2–3 diaminophenazine	Bluish green	Hydrothermal	3.2	0.91	[21]
Pollen	-	Blue	Hydrothermal	2.01	7.7	[24]
Citric acid monohyrdrate	o-phenylenediamine	Blue	Hydrothermal	3.0 ± 0.8	92.1	[47]
Grapehene	Citric acid and dicyanamide	Blue	Hydrothermal	2.3	36.5	[48]
Anhydrous gadolinium chloride (GdCl_3_)	3,4-dihydroxyhydrocinnamic acid (DHCA), 2,2′-(ethylenedioxy)bis(ethylamine) (EDA),	Blue	Hydrothermal	2.58	N/A	[49]
Aconitic Acid	N/A	Blue	Hydrothermal and microwave assisted	N/A	45.1–56.5	[84]
Citric Acid Glutathione in Formamide	Nitrogen, sulphur, boron, fluorine	Blue, Green and Red	Microwave mediated pyrolysis	6.1–10.0	22.9	[88]

## Data Availability

Not applicable.
